# Amino Acid Metabolism in Chronic Liver Disease: from Pathogenic Driver to Therapeutic Target

**DOI:** 10.7150/ijbs.128644

**Published:** 2026-03-25

**Authors:** Yingchen Huang, Yao Jiang, Wenkai Ye, Xin Hu, Nuerbiye Abudurexiti, Jiarui Li, Chuang Li, Ronggao Chen, Xiao Xu

**Affiliations:** 1 The fourth School of clinical Medicine, Zhejiang Chinese Medical University, Hangzhou First People's Hospital, 310006, China.; 2 School of Basic Medical Sciences and Forensic Medicine, Hangzhou Medical College, Hangzhou, Zhejiang, China, 311399, China.; 3 Zhejiang Chinese Medical University, Hangzhou, Zhejiang, 310051, China.; 4 Zhejiang University School of Medicine, Hangzhou, 310058, China.; 5 Department of Hepatobiliary and Pancreatic Surgery, The First Affiliated Hospital, Zhejiang University School of Medicine, Hangzhou, 310006, China.; 6 Hepatobiliary Center, The First Affiliated Hospital of Nanjing Medical University, Nanjing, 210029, China.; 7 NHC Key Laboratory of Combined Multi-organ Transplantation, Hangzhou, 310003, China.

**Keywords:** amino acid metabolism, chronic liver disease, MASLD/MASH, HCC

## Abstract

Amino acid metabolism is central to the liver's multifaceted physiology, serving as the cornerstone for protein homeostasis, metabolic integration, and tissue repair and regeneration. In addition, the dysregulation of amino acid metabolism is intricately linked to the pathogenesis and progression of a wide spectrum of liver diseases, acting as a central pathological driver beyond a passive metabolic consequence. In metabolic dysfunction-associated steatotic liver disease (MASLD), characteristic alterations in circulating branched-chain amino acids (BCAAs) and glycine levels directly promote hepatic steatosis, oxidative stress, and inflammation. The progression to hepatocellular carcinoma (HCC) is fueled by a profound metabolic reprogramming that creates a dependency on amino acids like glutamine and aspartate for energy and biomass, while methionine and tryptophan metabolism foster an immunosuppressive microenvironment and epigenetic dysregulation to facilitate immune evasion and tumor growth. Furthermore, in liver fibrosis and cirrhosis, metabolic adaptations support disease progression, whereas in hepatic encephalopathy, the hallmark imbalance between BCAAs and aromatic amino acids, coupled with ammonia neurotoxicity, disrupts neurotransmitter balance. These disease-specific alterations not only provide robust biomarkers for diagnosis and prognosis but, more importantly, reveal critical therapeutic vulnerabilities. Consequently, targeting amino acid metabolism emerges as a promising strategic avenue, encompassing dietary interventions, targeted supplementation, and pharmacological inhibition for the development of novel therapeutics across the landscape of liver diseases. This review aims to systematically expound on these dual physiological and pathological roles, arguing that such disease-specific metabolic alterations not only provide biomarkers but, more importantly, unveil targetable vulnerabilities, thereby positioning amino acid metabolism as a strategic frontier for developing novel therapeutics across liver diseases.

## 1. Introduction

The liver occupies a unique and dual-functional position within the human physiological system. Functioning as a central metabolic hub, it orchestrates the metabolism of key nutrients, including glucose, lipids, and amino acids 1. Moreover, the liver acts as a pivotal immune organ, harboring diverse immune cell populations and playing a critical role in host defense against pathogens 2. Amino acids, which are defined as organic compounds containing both amine and carboxylic acid functional groups, serve as the fundamental building blocks for protein synthesis in cellular metabolism 3. In addition, amino acids serve as intermediate metabolites that affect the biosynthesis of lipids, glutathione, nucleotides, glucosamine, and polyamines, as well as cell proliferation and tricarboxylic acid cycle carbon flux 4,5. The liver contains a rich pool of amino acids, including abundant non-essential types—such as alanine, aspartate, glutamate, glycine, and serine—as well as essential amino acids like histidine and threonine 5. Importantly, amino acids function not only as substrates for protein synthesis but also as key regulators in maintaining the homeostasis of the hepatic microenvironment, mediated by both hepatocytes and non-parenchymal cells 6, 7.

A growing body of recent evidence underscores that disruptions in amino acid metabolism within the hepatic microenvironment are closely linked to the onset and progression of a spectrum of chronic liver diseases, including metabolic dysfunction-associated steatotic liver disease (MASLD), hepatocellular carcinoma (HCC), liver failure and pathological processes such as liver fibrosis, regeneration 8,9,10. Consistent with this, clinical studies reveal that patients with chronic liver disease consistently display characteristic alterations in their plasma amino acid profiles. For instance, circulating amino acid levels may exhibit a potential causal relationship with MASLD risk; however, the underlying pathophysiological mechanisms remain to be fully elucidated 10. A hallmark of HCC progression is the loss of tyrosine catabolism, which results in elevated serum tyrosine levels and is frequently associated with the inhibition of the enzyme 4-hydroxyphenylpyruvate dioxygenase (4-HPD) 11. Similarly, large-scale metabolomic studies on NAFLD have demonstrated that alterations in specific amino acids, such as glutamic acid and glycine, are independently associated with the severity of liver fibrosis 9. Furthermore, patients with compensated liver disease exhibit a characteristic imbalance in the ratio between aromatic amino acids (AAAs) and branched-chain amino acids (BCAAs) 12. Consequently, the serum ratio of branched-chain amino acids to tyrosine (BCAA/Tyr) has been demonstrated to hold significant prognostic value for patients with cirrhosis 13.

Moving beyond their metabolic roles, amino acids—once considered primarily as nutrients—are now recognized as pivotal signaling molecules integral to a wide range of biological processes. Cells meticulously modulate critical functions—including protein synthesis and degradation, homeostasis maintenance, and cell fate decisions—by sensing fluctuations in both intracellular and extracellular amino acid concentrations 14. A prime example is the mTORC1 kinase, a master regulator of cell growth, whose activity is synergistically controlled by the integration of amino acid and growth factor signaling pathways 15,16. This regulatory function is particularly evident in tumor biology, where amino acid metabolic reprogramming critically influences cancer cell proliferation, survival, and interactions with the immune system. For instance, the HIF-1 signaling pathway drives tumor progression by coordinately regulating diverse metabolic pathways, including those for carbohydrates, nucleotides, lipids, and amino acids 17. Beyond systemic and tumorigenic contexts, enteric endocrine cells (EECs) directly sense dietary amino acids via specific receptors, leading to the secretion of regulatory peptides that modulate feeding behavior 18. In summary, this paradigm shift—from viewing amino acids solely as nutrients to recognizing them as integral signaling molecules—provides a novel conceptual framework for understanding chronic liver disease pathogenesis and opens promising avenues for therapeutic intervention.

## 2. Amino acid metabolic networks in the liver

### 2.1 Hepatocytes

Hepatocytes efficiently take up the majority of amino acids (with the exception of branched-chain amino acids) via specific transporters, a process that underpins the liver's central role in maintaining systemic amino acid homeostasis 19. Specific transporters, including SLC7A2 and SLC38A2, are critical for this catabolic function, and their expression is itself regulated to fine-tune hepatic amino acid uptake 20. This regulatory layer is further exemplified by RAP1A, which, upon activation, promotes the internalization of membrane-bound amino acid transporters, thereby reducing hepatic amino acid uptake and subsequent mTORC1 signaling pathway activation 21. Following uptake, amino acids are catabolized within hepatocytes, yielding products such as urea, gluconeogenic precursors, and other bioactive molecules. Amino acid stimulation triggers a coordinated response in hepatocytes, enhancing gluconeogenesis and ureagenesis while concurrently activating AMPK through an ASS1-dependent mechanism, thereby modulating energy metabolism 22. Beyond their catabolic roles, certain amino acids like glutamine serve as key nitrogen donors in hepatic mitochondrial metabolism and replenish metabolic intermediates through anaplerotic reactions. Notably, dysregulation of glutamine metabolism in liver disease states can trigger significant metabolic reprogramming 23. In summary, hepatocytes demonstrate remarkable plasticity and reprogramming capacity within their intricate amino acid metabolic networks. This inherent plasticity is essential for maintaining hepatic homeostasis through the coordinated regulation of amino acid, lipid, and glucose metabolism. Consequently, pathological conditions—including steatosis, chronic liver disease, and HCC—disrupt these finely tuned mechanisms, culminating in systemic metabolic dysregulation.

### 2.2 Non-parenchymal cells (NPCs)

**Figure 1:**
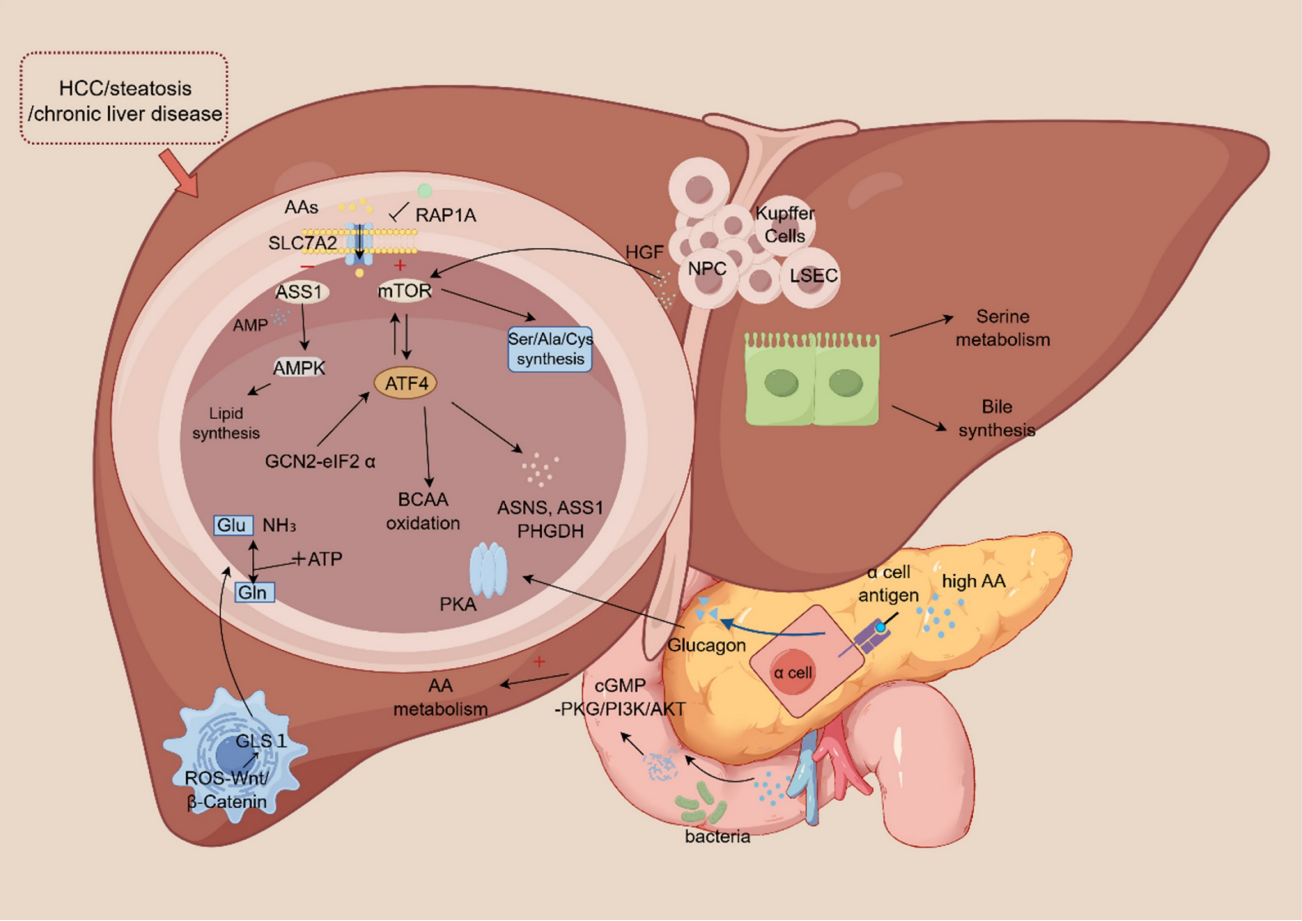
Schematic illustration of the hepatic amino acid metabolic network, integrating roles of hepatocytes, non-parenchymal cells (NPCs), and systemic regulators. In hepatocytes, amino acid uptake via transporters (e.g., SLC7A2, SLC38A2) fuels catabolism and anabolism, regulated by RAP1A and the ASS1-AMPK axis. Key regulatory hubs include the mTORC1-ATF4 positive feedback loop, which coordinates amino acid flux and synthesis, and the GCN2-eIF2α-ATF4 pathway, activated by amino acid deprivation. NPCs, including hepatic stellate cells (HSCs), cholangiocytes, and endothelial cells, contribute via paracrine signaling and specialized metabolism. Systemic integration occurs via the liver-pancreas axis, where hyperaminoacidemia stimulates glucagon release, activating hepatic PKA signaling to enhance catabolism. Gut microbiota-derived metabolites modulate hepatic metabolism through cGMP-PKG and PI3K/AKT pathways. Disruption of this network underlies pathologies such as steatosis, chronic liver disease, and HCC.

Beyond the central role of hepatocytes, NPCs are increasingly recognized as crucial contributors to the hepatic amino acid metabolic network. For instance, hepatic stellate cells (HSCs) modulate amino acid metabolism in hepatocytes in a paracrine manner by secreting signaling molecules like R-spondin 3 and hepatocyte growth factor 24. A specific example is that HSCs upregulate glutaminase 1 (GLS1) expression through the ROS-Wnt/β-Catenin pathway, thereby promoting glutamine catabolism. This function is distinct from hepatocytes, which predominantly express the GLS2 isoform 25. Similarly, cholangiocytes participate in serine metabolism and contribute to bile acid synthesis, functions that are clearly distinct from the urea cycle activities predominant in hepatocytes 26. Likewise, endothelial cells utilize glycine metabolism to support immune regulation and maintain vascular barrier integrity, highlighting a metabolic specialization separate from the energy-producing pathways central to hepatocytes 27. The functional interplay between NPCs and hepatocytes is particularly evident during liver regeneration. In this process, hepatocyte growth factor (HGF) secreted by NPCs activates the mTORC1-ATF4 axis in hepatocytes, driving the synthesis of alanine, aspartate, and methionine 28. Furthermore, under conditions of disrupted hepatocyte lipid metabolism, HSCs can compensate for the energy deficit by enhancing their own glutamine metabolism 29. Single-cell RNA sequencing studies provide further evidence of metabolic rewiring in disease states. For example, in fatty liver disease, HSCs upregulate genes involved in lipid metabolism, whereas hepatocytes downregulate the amino acid transporter SLC7A2 20. This integrated regulatory network within the liver lobule is depicted in Figure 1.

### 2.3 Integrated regulatory network

The mammalian target of rapamycin (mTOR) is a central serine/threonine kinase that plays a master regulatory role in cell growth and metabolism 30. The mTORC1 is particularly sensitive to fluctuations in amino acid availability, integrating intracellular signals derived from amino acids to coordinately control the metabolism of amino acids, glucose, nucleotides, and lipids 31. For instance, in hepatocytes, insulin-stimulated mTORC1 activation promotes the synthesis of specific amino acids, including serine, alanine, and cysteine 28. Beyond transcriptional regulation, mTORC1 also modulates amino acid metabolism through post-translational mechanisms, such as phosphorylating downstream effectors to inhibit the activity of the cystine/glutamate antiporter (system xc-) 32. Activating transcription factor 4 (ATF4) is a central transcriptional regulator of the integrated stress response (ISR) 33. Under conditions of amino acid deprivation or general nutritional stress, ATF4 expression is primarily induced via the GCN2-eIF2α axis, wherein phosphorylation of eIF2α serves as the pivotal upstream event triggering its translation 34. Upon activation, ATF4 transcriptionally upregulates a suite of genes encoding amino acid transporters (e.g., SLC1A5) and metabolic enzymes (e.g., ASNS, ASS1, PHGDH) to sustain intracellular amino acid pools and redox homeostasis 35. mTOR and ATF4 constitute a core regulatory axis that often operates through a positive feedback loop to amplify amino acid metabolic flux, mTORC1 signaling can enhance ATF4 expression 36. In turn, ATF4-upregulated amino acid transporters increase nutrient uptake, which sustains mTORC1 activation, thereby creating a self-reinforcing cycle that fuels cell growth and anabolism 37. However, under distinct stress conditions, ATF4 can be activated independently of mTORC1 primarily through the GCN2-eIF2α pathway 38. This mTORC1-independent ATF4 activation drives a metabolic reprogramming adaptive for survival, characterized by increased branched-chain amino acid oxidation and glutamine utilization, to maintain metabolic flexibility and ensure cell viability 38.

An increase in amino acid flux initiates a metabolic cascade in which argininosuccinate synthase (ASS) catabolizes arginine, generating AMP—a potent activator of AMPK 39. This ASS-AMPK axis not only promotes amino acid degradation but also suppresses lipogenic pathways, thereby coupling amino acid catabolism to the regulation of hepatic lipid metabolism 22. Hepatic pathological states, such as metabolic dysfunction-associated steatohepatitis (MASH), are characterized by inflammatory signaling that profoundly reprograms amino acid metabolism 40. Beyond these, the liver engages in intricate feedback loops with the endocrine system to coordinate systemic metabolism. A prime example is the liver-pancreas axis: glucagon secretion from pancreatic α-cells is stimulated by elevated amino acid levels, while glucagon, in turn, signals to the liver to enhance amino acid catabolism 41. Specifically, hyperaminoacidemia (e.g., elevated branched-chain amino acids) stimulates glucagon release. This hormone then activates the cAMP-dependent protein kinase (PKA) signaling cascade in the liver, promoting hepatic amino acid catabolism 42. Conversely, in conditions like hepatic steatosis, this finely tuned feedback loop is disrupted, leading to dysregulated hepatic amino acid metabolism^19^. The integrity of this liver-endocrine communication is particularly critical in metabolic syndrome, as it serves as a key link between hepatic metabolic function and systemic endocrine homeostasis 19, 28.

Gut microbiota also regulated hepatic amino acid metabolism through metabolite signaling for example, aromatic amino acids (such as tryptophan and phenylalanine) undergo bacterial degradation to produce metabolites like indole and anthranilic acid, which activate cGMP-PKG and PI3K/AKT pathways 43. These signaling molecules modulate hepatic amino acid synthesis (e.g., alanine metabolism) and immune responses.

The regulation of hepatic amino acid metabolism constitutes a sophisticated, multi-layered network that integrates inputs from hepatocytes, NPCs, energy stress, and gut microbiota. This intricate orchestration, mediated by transcription factors, kinases, metabolic sensors, and transporters, enables the liver to dynamically adapt amino acid flux in response to nutritional fluctuations, stress challenges, and pathological insults. Elucidating how disruptions in amino acid metabolism contribute to the pathogenesis and progression of liver diseases has therefore emerged as a critical frontier in biomedical research, offering novel insights into the therapeutic intervention.

## 3. Amino acid metabolism in liver physiology and chronic liver disease

### 3.1 MASLD

In accordance with the latest nomenclature, the term “fatty liver disease” in this review refers to MASLD and its progressive form, MASH. This updated terminology supersedes the previous designations of NAFLD and non-alcoholic steatohepatitis (NASH). Almost all patients with NAFLD meet the diagnostic criteria for MASLD, and their natural histories are essentially consistent 44. Metabonomic and transcriptomics analysis showed that branched chain amino acid degradation, glycine synthesis, and glutamine metabolism pathways were significantly dysregulated in fatty liver, interacting with fatty acid metabolism and exacerbating liver lipid accumulation 45,46,47. Consequently, amino acids are not merely biomarkers but also active contributors to disease pathogenesis. Serving as important carbon sources for de novo lipogenesis, they directly promote hepatic steatosis, oxidative stress, and inflammation through multiple mechanisms, thereby accelerating the progression from steatosis to steatohepatitis 48.

#### 3.1.1 BCAAs

Amino acid metabolism is commonly dysregulated in fatty liver disease, a state characterized by elevated circulating levels of BCAAs and reduced glycine. This metabolic perturbation has been recognized by numerous studies as a potential biomarker or pathogenic factor in the disease 49,50. Notably, BCAA concentrations undergo dynamic changes during fatty liver progression. In the early disease stage (MASLD), circulating BCAAs are significantly elevated, and this increase is linked to heterogeneity in hepatocyte lipid droplet size and an elevated risk of diabetes. However, as the disease progresses to MASH or cirrhosis, circulating BCAA levels decline rapidly 51. Thus, BCAAs exhibit a context-dependent dual role, and their impact may shift from detrimental to protective during the progression from MASLD to MASH. On one hand, BCAA supplementation can significantly reduce hepatic triglyceride content and lipid droplet area by inhibiting the expression and protein levels of fatty acid synthase (FASN), thereby alleviating hepatic steatosis and liver damage in NASH via activation of the mTOR pathway 52,53. Consistently, in an egg-laying chicken model, a high-BCAA diet was shown to promote ketosis and energy metabolism by inhibiting the tryptophan-ILA-AHR axis and MAPK9-mediated de novo lipogenesis. It also activates PPAR-RXR signaling and pexophagy to enhance fatty acid β-oxidation, thereby alleviating NAFLD 54. On the other hand, In terms of BCAA metabolism, leucine, a ketogenic amino acid, can generate acetyl-CoA via conversion to acetoacetate, supplementing the deficiency of acetyl-CoA derived from pyruvate and participating in hepatic de novo lipogenesis 55. This process is also one of the mechanisms through which BCAAs promote lipid accumulation in specific contexts. Conversely, BCAAs can also exacerbate hepatic oxidative stress and hepatocyte apoptosis. For instance, although BCAAs reduce hepatic fat deposition in HFD-fed mice, they concurrently increase hepatic oxidative stress, hepatocyte apoptosis, and circulating liver enzyme levels, ultimately inducing liver damage 53. This detrimental effect is associated with mTOR overactivation. Specifically, mTOR overactivation inhibits hepatic autophagy and heightens hepatocyte susceptibility to free fatty acid-mediated lipotoxicity 53. The regulation of amino acid metabolism involves several key pathways. Among them, the GCN2-ATF4 pathway is activated under amino acid starvation: GCN2 senses uncharged tRNAs and activates the transcription factor ATF4, which in turn upregulates the expression of amino acid transporters such as SLC3A2 and SLC7A5 56,57.

Notably, the gut microbiota serves as a crucial contributor to amino acid metabolic disorders. For example, humans lack the capacity for de novo BCAA synthesis, whereas gut bacteria such as Prevotella copri and Bacteroides vulgatus can synthesize BCAAs. This microbial synthesis can exacerbate insulin resistance and thereby promote fatty liver progression 58. Furthermore, a reduction in Bacteroides thetaiotaomicron abundance elevates circulating levels of glutamate, BCAAs, and AAAs. Conversely, restoring this bacterial population via weight loss or direct supplementation lowers these amino acid levels and ameliorates obesity-related metabolic dysfunction 59. A direct causal role was demonstrated by a study in which CRISPR-mediated knockout of the BCAA transaminase BCAT gene in Clostridium sporogenes increased host serum BCAA concentrations yet concurrently improved glucose tolerance 60. This finding indicates that gut microbiota-derived amino acid metabolism directly shapes the host's hepatic metabolic status.

#### 3.1.2 Glycine

Glycine deficiency fuels the progression of MASLD by compromising both antioxidant defense systems and lipid catabolic processes. Consistently, low glycine levels have been identified as a biomarker for NAFLD and are posited to drive disease progression by impairing fatty acid oxidation and glutathione (GSH) synthesis. Glycine is one of the key precursors for the synthesis of glutathione. Consequently, in the setting of hepatic steatosis, diminished glycine availability results in insufficient GSH production. This deficit exacerbates oxidative stress, thereby accelerating disease progression 61. Beyond its role in antioxidant defense, glycine also participates in one-carbon metabolism. For instance, patients with hepatic steatosis frequently exhibit downregulation of glycine N-methyltransferase (GNMT). This downregulation leads to accumulation of S-adenosylmethionine (SAMe), which in turn promotes hepatic steatosis 62. The SAMTOR-mTOR pathway represents another regulatory axis. In this pathway, SAMe activates mTORC1 via the sensor SAMTOR, thereby promoting lipogenesis through the transcription factor sterol regulatory element-binding protein (SREBP) 63. Conversely, aberrant methionine metabolism that results in SAMe deficiency can inhibit this pathway and exacerbate liver injury. The absence of serine hydroxymethyltransferase 2 (SHMT2) increases circulating glycine levels but reduces hepatic methylation potential, thereby enhancing susceptibility to hepatic steatosis 64. Glycine deficiency inhibits fatty acid β-oxidation in mitochondria and peroxisomes, thereby exacerbating hyperlipidemia and steatohepatitis induced by a high-fat diet (HFD) 65. In patients with hepatic steatosis, glycine deficiency not only inhibits fatty acid β-oxidation in mitochondria and peroxisomes but also reduces the conversion of the glycolytic intermediate 3-phosphoglycerate (3-PG) to serine. Simultaneously, glycine deficiency decreases the supply of carbon sources for the tricarboxylic acid (TCA) cycle, further exacerbating the imbalance between hepatic gluconeogenesis and hepatic lipogenesis 66.

#### 3.1.3 Glutamine

**Figure 2:**
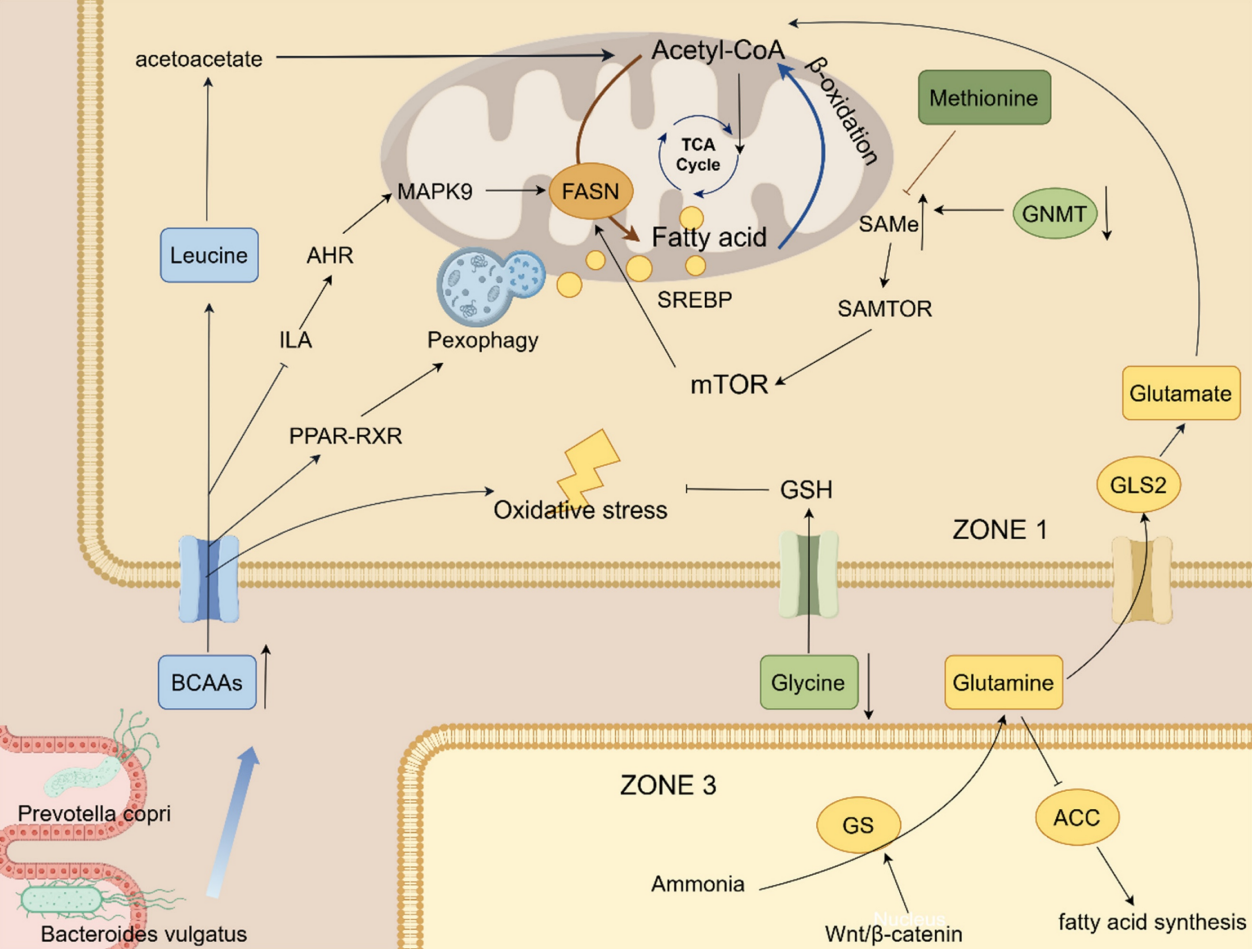
This schematic diagram illustrates the core amino acid metabolic networks and regulatory pathways implicated in the development and progression of MASLD/MASH. Key amino acids (BCAAs, leucine, glycine, glutamine, glutamate, methionine) modulate hepatic lipid metabolism, oxidative stress, and energy homeostasis through multiple mechanisms: BCAAs can generate acetyl-CoA via β-oxidation or regulate fatty acid synthesis (mediated by FASN) and catabolism through pathways such as mTOR, PPAR-RXR, and MAPK9; glycine deficiency exacerbates oxidative stress by impairing glutathione (GSH) synthesis, while its metabolite SAMe regulates lipogenesis via the SAMTOR-mTOR-SREBP axis; glutamine metabolism exhibits distinct zonation within the hepatic lobule—mitochondrial GLS2 in the periportal region (ZONE1) hydrolyzes glutamine into glutamate and ammonia (supporting the TCA Cycle, energy production, and gluconeogenesis), whereas GS (regulated by the Wnt/β-catenin signaling pathway) in the perivenous region (ZONE3) synthesizes glutamine from residual ammonia to maintain systemic nitrogen balance. Additionally, gut microbiota (e.g., Prevotella copri, Bacteroides vulgatus) can synthesize BCAAs to indirectly influence host hepatic metabolism. Collectively, key enzymes (ACC, GNMT) and biological processes (pexophagy, fatty acid synthesis) constitute a core regulatory network through which amino acid metabolic dysregulation drives fatty liver progression.

Accumulating evidence suggests that glutamine exerts protective effects against hepatic steatosis. Glutamine metabolism exhibits distinct zonation within the hepatic lobule: in the periportal region (zone 1) of the hepatic lobule, mitochondrial glutaminase 2 (GLS2) dominates the hydrolysis of glutamine into glutamate and ammonia 67. Ammonia is detoxified via the urea cycle, while glutamate enters the TCA cycle to support energy production and provides substrates for gluconeogenesis in this zone. Conversely, in the perivenous region (zone 3), glutamine synthetase (GS)—whose expression is regulated by the Wnt/β-catenin signaling pathway—catalyzes glutamine synthesis from residual ammonia within hepatocyte cytoplasm, thereby maintaining systemic nitrogen balance. Furthermore, glycolysis is predominant in this hypoxic zone and acts synergistically with glutamine metabolism to support hepatic lipogenesis. In the context of MASH, GLS2 expression is downregulated, while GLUL (encoding GS) expression is concurrently upregulated. This altered expression profile elevates the glutamate/glutamine ratio in peripheral blood, serving as a potential disease biomarker 68. Meanwhile, chronic hypoxia induces the activation of GLS1 in HSCs, accelerating glutaminolysis and promoting fibrosis progression. Experimentally, glutamine supplementation has been shown to inhibit key hepatic lipogenic enzymes such as acetyl-CoA carboxylase (ACC), thereby reducing fatty acid synthesis and hepatic lipid accumulation 69. Moreover, glutamine also ameliorates hepatic steatosis by regulating the PPAR-mediated amino acid transport pathway and optimizing fatty acid utilization 69. Similarly, L-carnitine has been demonstrated in experiments to protect mice from the development of NAFLD through antioxidant and lipid metabolism reprogramming, but the specific mechanism still needs to be clarified 70. A schematic overview of these interconnected metabolic perturbations in MASLD is provided in Figure 2.

In summary, the dysregulation of BCAAs, glycine, and glutamine represents a core pathological axis in MASLD/MASH, driving disease progression by dysregulating lipogenesis, exacerbating oxidative stress, and disrupting inter-organ signaling. The context-dependent duality of metabolites such as BCAAs, coupled with the spatial reorganization of glutamine metabolism during disease progression, underscores the complexity of this metabolic network 71. While amino acid metabolism presents promising therapeutic targets, future research must prioritize understanding the dynamic interactions within these pathways and with the gut microbiome. Such efforts are essential for translating these fundamental discoveries into precise diagnostic biomarkers and effective, stage-specific therapeutic strategies for patients.

### 3.2 HCC

Reprogramming of amino acid metabolism is a central hallmark of HCC, primarily supporting the malignant phenotype by providing energy, synthesizing macromolecules, and maintaining redox homeostasis. Furthermore, amino acid metabolism collaborates with carbohydrate and lipid metabolism to jointly establish the integrated metabolic network characteristic of HCC.

#### 3.2.1 Glutamine and glutamate

Glutamine serves as a fundamental nutrient for HCC, fuelling anabolic growth and maintaining redox homeostasis. To support this demand, HCC cells avidly take up exogenous glutamine through transporters such as ASCT2 and SNAT1 (SLC1A5/SLC38A1) 72,73. Within the cell, glutamine is deamidated to glutamate by glutaminase (GLS). The resulting glutamate acts as a pivotal metabolic node, as it can be converted to α-ketoglutarate (α-KG) by glutamate dehydrogenase (GLUD) or transaminases. This reaction replenishes the TCA cycle for energy production and generates NADPH to support biosynthesis and antioxidant defense 74,75. This metabolic reprogramming is reinforced by oncogenic signaling. For example, the Wnt/β-catenin pathway upregulates GS to boost de novo glutamine synthesis 76,77. Conversely, downregulation of glutamate-oxaloacetate transaminase 2 (GOT2) enhances glutaminolytic flux, thereby driving proliferation through activation of the PI3K/AKT/mTOR pathway 78,79,80.

Beyond its role as a metabolic intermediate, glutamate functions as a critical signaling molecule. For instance, overexpression of glutamate and its receptor NMDA Receptor Subunit 2B in HCC promotes tumor invasion by activating the calcium-dependent PLC-γ/PKC pathway 81,82. Furthermore, glutamate signaling through the overexpressed NR2B promotes C-C motif chemokine ligand 2 (CCL2) expression by suppressing the histone methyltransferase EZH2. This reduction in EZH2 decreases repressive H3K27me3 marks on the CCL2 promoter, leading to CCL2 upregulation. The elevated CCL2, in turn, enhances recruitment of tumor-associated macrophages, thereby fostering tumor progression 83. Under nutrient stress, glutamate supports HCC proliferation by promoting macropinocytosis, an adaptive pathway that provides energy and biosynthetic precursors 84,85. Notably, GLUD1 silencing exerts a dual effect: it not only perturbs glutamate catabolism but also reduces α-KG levels, leading to HIF-1α stabilization and enhanced hypoxic survival 86,87.

Aspartate biosynthesis relies on glutamine-derived α-KG. Intriguingly, when ASS1 is inhibited by lncRNA LINC01234, the resulting aspartate accumulation does not directly activate mTOR but instead promotes glutamate uptake by upregulating the SLC1A3 transporter, forming a feedforward “aspartate-glutamate-mTOR” activation loop. Conversely, GOT2 deletion reduces aspartate levels but compensates by enhancing glutaminolysis, thereby increasing the dependency on GLS and sensitizing HCC cells to GLS inhibitors 88.

Reprogrammed proline metabolism supports HCC proliferation and confers drug resistance. This reprogramming is characterized by upregulation of PYCR1 and ALDH18A1 and downregulation of PRODH. Consequently, proline accumulation provides precursors for extracellular matrix (ECM) production, and activation of the AKT pathway upregulates epithelial-mesenchymal transition (EMT) transcription factors 89. High expression of ALDH18A1 reduces the sensitivity of sorafenib, and its inhibitor can increase the killing rate of drug by 37% 90.

#### 3.2.3 Methionine, lysine and tryptophan

Methionine, arginine, lysine and tryptophan are the cross hubs of immune escape and epigenetic reprogramming, which regulate HCC progression through the cascade of metabolites, epigenetic modifications and immune function.

Methionine plays a complex dual role in HCC, its dysregulated metabolism serves not only as a metabolic hallmark of tumor cells but also presents a potential target for immunotherapy. On the one hand, tumor cells are metabolically dependent on methionine, and abnormal activation of its metabolic cycle is one of the hallmarks of HCC 91. Upregulation of methionine adenosyltransferase 2A (MAT2A) accelerates the methionine cycle, promoting the production of SAMe. SAMe, in turn, drives tumor progression by facilitating histone methylation 92. The oncogene c-Myc enhances MAT2A expression by promoting the degradation of SIRT4, thereby amplifying the oncogenic output of methionine metabolism 92. High concentrations of methionine activate AMPK and mTOR pathways and promote the proliferation and migration of hepatocellular carcinoma cells. Inhibition of AMPK attenuated the cancer-promoting effect of methionine 93. On the other hand, L-methionine significantly enhanced the killing effect of CD8+ T cells on HCC cells by inhibiting NR1I2/PCSK9 signaling. Mechanistically, L-methionine downregulates the transcription factor NR1I2, which in turn suppresses PCSK9 expression and relieves its inhibitory effect on immune cells 94. Consistent with this therapeutic potential, methionine deprivation induces irreversible cell cycle arrest in hepatoma cells, highlighting methionine restriction as a promising therapeutic strategy 95. 3M-Gel, a methionine metabolism regulator, induces immunogenic death of tumor cells and activates anti-tumor immune responses by limiting SAM production and histone methylation 96.

Arginine metabolism is highly active in HCC, and this activity promotes tumor formation via altering the expression of metabolic genes 97. The uptake of exogenous arginine via the transporter SLC7A1 activates the mTORC1/S6K1 pathway in HCC cells. Intriguingly, this activation is modulated by TM4SF5-mediated arginine efflux 98,99. Consequently, inhibition of SLC7A1 impairs HCC cell growth 100. Argininosuccinate synthase 1 (ASS1) expression is heterogeneous in HCC. At low levels, tumors become auxotrophic for exogenous arginine. Conversely, high ASS1 expression enables co-generation of nitric oxide with arginine, which inhibits the PI3K/Akt pathway and enhances cisplatin sensitivity 101, 102. Alcohol consumption inhibits protein arginine methyltransferase 1 (PRMT1), thereby disrupting arginine methylation patterns, which is a proposed mechanism for alcohol-associated HCC promotion 103. PRMT3 is highly expressed in HCC, and its overexpression promotes tumor cell proliferation and metastasis, while knockdown inhibits these effects 104. Additionally, the hepatitis B virus-encoded HBx protein modulates ferroptosis by regulating PRMT9, thereby contributing to HCC progression 105.

Lysine influences HCC progression through multifaceted mechanisms, including metabolic competition, epigenetic modification, and immune regulation 106. HCC cells competitively uptake lysine from the microenvironment through the high expression of lysine transporter SLC3A2, resulting in a lack of lysine in T cells, thereby inhibiting the activation and function of T cells 106. Mechanistically, lysine deficiency reduces STAT3 levels in T cells, thereby suppressing their proliferation and effector functions and ultimately promoting tumor progression 106. Multiple types of lysine modifications are dysregulated in HCC, constituting a complex regulatory network. Succinyl-CoA dehydrogenase affects the progression of HCC by regulating lysine crotonylation 107. In addition, SIRT3, an NAD+-dependent deacetylase, is lowly expressed in HCC, and its deficiency leads to an increase in non-histone lysine lactylation levels, promoting tumor development 108. Lysine acetylation also regulates the stability of the p62 protein. This modification is dynamically controlled by the opposing activities of Sirt1 (deacetylase) and GCN5 (acetyltransferase), thereby influencing HCC cell growth 109. The lysine succinylation regulated by SIRT5 is abnormal in HCC and may promote tumor progression by affecting the activity of metabolic enzymes (such as citrate synthase CS) 110. Furthermore, the histone lysine methyltransferase SETD1A contributes to sorafenib resistance, suggesting that targeting SETD1A could improve therapeutic outcomes in HCC 111.

Tryptophan metabolism is a key pathway for the formation of HCC immunosuppressive microenvironment. The enzymes indoleamine 2,3-dioxygenase 1 (IDO1) and tryptophan 2,3-dioxygenase (TDO2) are upregulated in HCC. Tryptophan catabolism through the kynurenine pathway generates kynurenine (Kyn), which activates the aryl hydrocarbon receptor (AhR). AhR activation inhibits CD8⁺ T cell function, impairs B cell differentiation into germinal center B cells, and disrupts tertiary lymphoid structure (TLS) function 112,113. The high expression of TDO2 is a key driver of TLS maturation defects. Consequently, Tdo2 knockdown improves the response to anti-PD-1 therapy 113.

#### 3.2.4 Other Amino acids

Beyond the amino acids previously discussed, serine, cysteine/cystine and BCAAs further contribute to HCC pathogenesis. They drive drug resistance, bolster antioxidant defenses, and remodel the tumor microenvironment(TME) through distinct metabolic reprogramming mechanisms, collectively underpinning the metabolic foundation of the malignant phenotype.

Serine metabolism is critical for the maintenance of redox homeostasis and proliferation in HCC, especially in drug-resistant cells. Notably, while PHGDH transcript levels remain largely unchanged in HCC, PRMT1-mediated methylation enhances its catalytic activity, thereby promoting serine production to fuel nucleotide and GSH synthesis 114. The regulation of this pathway is complex: NRF2 directly binds to the PHGDH promoter to upregulate its expression, whereas the E3 ligase FBXO7 indirectly suppresses PHGDH activity by mediating the ubiquitination and degradation of PRMT1 81. Furthermore, high PHGDH activity increases NADPH production via the one-carbon metabolism pathway, thereby enhancing the antioxidant capacity of sorafenib-resistant cells. Consequently, pharmacological inhibition of PHGDH by NCT-503 resensitized resistant cells to sorafenib by 4.3-fold 115.

Cystine uptake and metabolism are the key barriers of HCC against ferroptosis. HCC cells import cystine via the xCT (SLC7A11) transporter, reduce it to cysteine, and utilize it for GSH synthesis. GSH, in turn, is used by glutathione peroxidase 4 (GPX4) to eliminate lipid peroxides, thereby inhibiting ferroptosis 116,117. While sorafenib partially inhibits xCT, this effect is counteracted in resistant cells through NRF2-mediated upregulation of xCT expression 118. Cysteine depletion not only induces ferroptosis but also activates caspase-1/GSDMD-N mediated pyroptosis 75.

**Figure 3:**
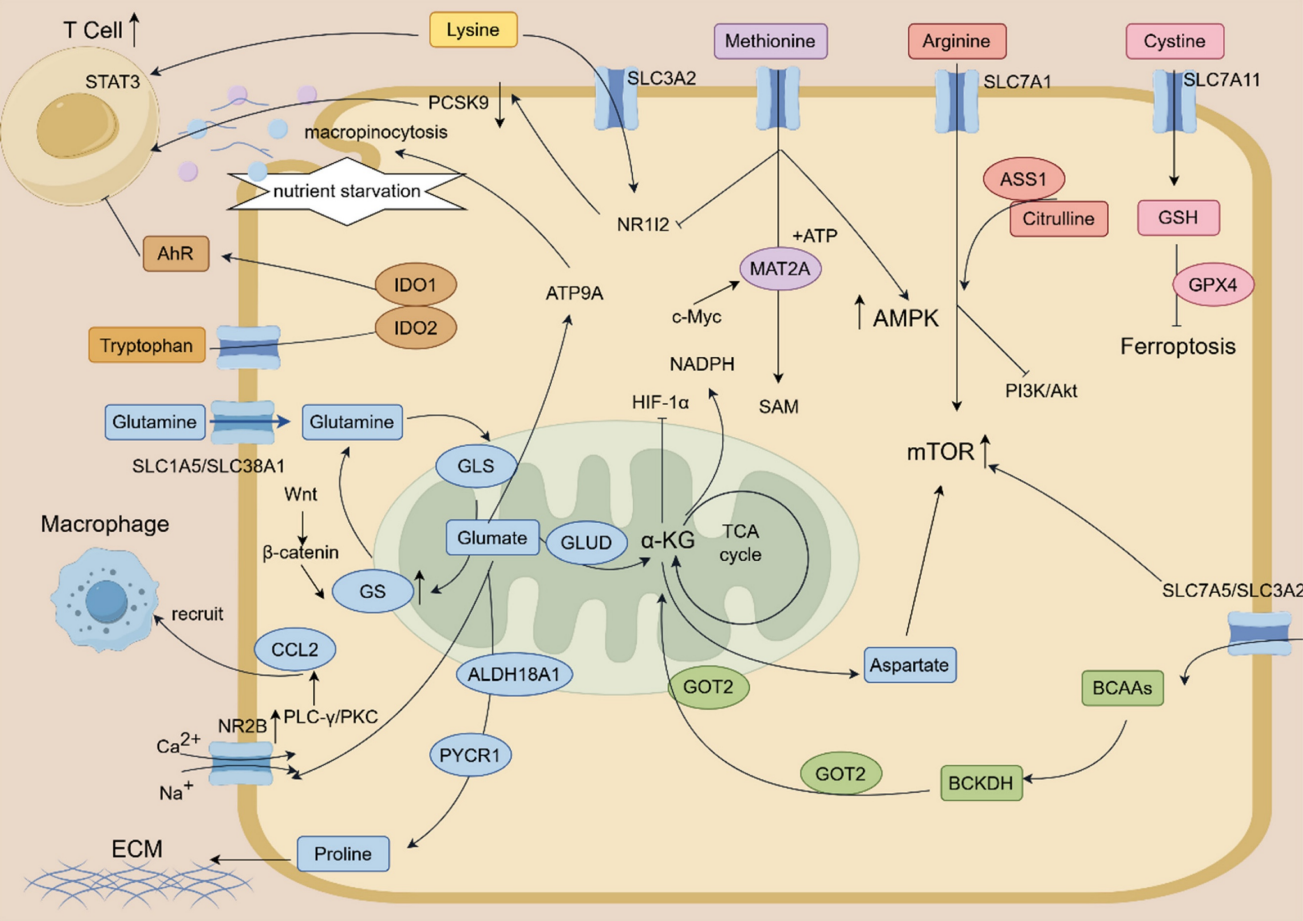
This schematic diagram depicts the core amino acid metabolic reprogramming and interconnected regulatory networks driving hepatocellular carcinoma (HCC) progression. Key amino acids (lysine, methionine, arginine, cystine, tryptophan, glutamine, aspartate, proline, BCAAs) modulate HCC malignant phenotypes. Glutamine is avidly taken up by HCC cells via SLC1A5/SLC38A1 transporters, and hydrolyzed to glutamate by GLS; glutamate further participates in the TCA cycle (via GLUD-mediated conversion to α-KG) or acts as a signaling molecule to activate calcium-dependent PLC-γ/PKC pathway (through NR2B) and promote CCL2 expression, thereby recruiting tumor-associated macrophages. Tryptophan catabolism (catalyzed by IDO1/IDO2) generates metabolites that activate AhR, suppressing T cell function and fostering an immunosuppressive microenvironment; methionine metabolism (regulated by MAT2A) produces SAMe to drive histone methylation, while also enhancing CD8+ T cell cytotoxicity via inhibiting NR1I2/PCSK9 signaling.Cystine uptake through SLC7A11 supports GSH synthesis, which collaborates with GPX4 to inhibit ferroptosis; BCAAs are transported via SLC7A5/SLC3A2 (LAT1) and regulate HCC survival under nutrient stress through AMPK/mTOR pathway or BCKDH-mediated catabolism to replenish the TCA cycle. Arginine (transported by SLC7A1) activates mTORC1/S6K1 signaling, with ASS1 expression dictating arginine auxotrophy or nitric oxide production; lysine uptake by HCC via SLC3A2 induces T cell lysine deficiency, reducing STAT3 levels and impairing T cell function. Oncogenic signaling pathways (Wnt/β-catenin, PI3K/Akt, HIF-1α, c-Myc) and key enzymes (GOT2, PYCR1, ALDH18A1, ACC) synergistically regulate amino acid metabolism, while processes such as macropinocytosis (under nutrient starvation) and proline-driven extracellular matrix (ECM) production further support HCC progression.

BCAA provides flexibility for HCC survival under nutrient stress. HCC cells import BCAAs primarily through the heterodimeric transporter LAT1 (SLC7A5/SLC3A2). Knockdown of LAT1 reduces BCAA uptake by 61% and downregulates mTORC1 signaling 119. High concentrations of BCAA activate the AMPK/mTOR pathway, and inhibition of AMPK attenuates its cancer-promoting effect 116. Furthermore, under glutamine starvation, PPM1K mediates the dephosphorylation and activation of BCKDHA. This enhances the catabolism of BCAAs to acetyl-CoA, thereby replenishing the TCA cycle. This adaptive metabolic shift can be therapeutically exploited, as it is blocked by the combination of BCKDHA and GLS inhibitors 120. The complex reprogramming of amino acid metabolism that fuels HCC progression is summarized in Figure 3.

In summary, amino acids orchestrate HCC progression through a interconnected network involving the mTOR pathway, metabolic reprogramming, and immunomodulation within the tumor microenvironment, collectively governing cancer cell proliferation, invasion, and immune evasion 121,122. This complexity underlies the context-dependent duality of amino acid functions in HCC, wherein the same metabolite can exert both pro-tumor and anti-tumor effects depending on the cellular and environmental context 123. Therefore, in subsequent studies, a comprehensive investigation of their regulatory mechanisms is of great significance for identifying these amino acid pathways as therapeutic targets and developing corresponding targeted strategies. 124.

### 3.3 Liver fibrosis and cirrhosis

In the development of liver fibrosis and cirrhosis, amino acid metabolic reprogramming plays multiple key roles, which not only participates in the systemic energy adaptation of the disease state, but also directly regulates the progression and reversal of fibrosis through specific mechanisms 125. In patients with acute-on-chronic liver failure (ACLF), blood metabolomic analyses reveal enhanced skeletal muscle catabolism. The resulting amino acids are funneled into high-energy anabolic processes, including nucleotide and protein synthesis, to meet the heightened energy demands of the disease state 126. Beyond their systemic metabolic roles, specific amino acids directly modulate the fibrotic process by regulating hepatic stellate cell activation, collagen metabolism, and mitochondrial function. For instance, L-serine exhibits anti-fibrotic potential by attenuating pathological collagen deposition 127. Similarly, L-aspartate treatment ameliorates liver fibrosis by reversing corticosterone (CORT)-induced activation of glucocorticoid receptor β (GRβ). This reversal alleviates the subsequent transcriptional repression of the mitochondrial genome, restoring mitochondrial function 128. In the context of glutamine metabolism, the mitochondrial sirtuin SIRT4 exerts anti-fibrotic effects by inhibiting the conversion of glutamate to α-ketoglutarate (α-KG). This inhibition curbs the proliferation of hepatic stellate cells (HSCs), thereby attenuating fibrosis 129.

### 3.4. Liver failure and hepatic encephalopathy (HE)

HE represents a major neurological complication of liver failure, the pathogenesis of which is intimately linked to severe disruptions in amino acid metabolism 130. In this process, decreased levels of BCAAs, an imbalance with aromatic amino acids AAAs, hyperammonemia, and glutamate-mediated neurotoxicity collectively constitute a core pathophysiological axis in HE 131. During liver failure, the levels of plasma BCAAs significantly decrease, which is closely associated with the occurrence of HE 132. This reduction in BCAAs disrupts the plasma BCAA/AAA ratio, which promotes the influx of AAAs across the blood-brain barrier. Consequently, AAA accumulation in the brain interferes with normal neurotransmitter synthesis 133. AAAs are precursors of neurotransmitters (such as 5-hydroxytryptamine, dopamine), and their excessive accumulation may trigger neurotoxicity 134. Hyperammonemia, resulting from the impaired hepatic clearance of ammonia, constitutes the central pathogenic factor in HE. During liver failure, the capacity for ammonia detoxification is compromised, leading to systemic hyperammonemia 135. Ammonia directly contributes to the neuropathology of HE by disrupting glutamatergic neurotransmission, inducing overactivation of N-methyl-D-aspartate (NMDA) receptors, and triggering astrocyte swelling 136. Furthermore, elevated cerebral glutamate levels are implicated in the excitatory neurotoxicity observed in HE. This neurotoxic effect is likely exacerbated by concomitant mitochondrial dysfunction and oxidative stress, culminating in progressive neuronal injury 137.

### 3.5 Liver Regeneration

The liver possesses a remarkable capacity for regeneration, a process in which amino acids serve as crucial energy sources and signaling molecules to orchestrate the underlying regulatory networks. Glutamine and glutamate metabolic network are the core energy and signal hub driving hepatocyte proliferation. For instance, glutamate reprograms macrophage metabolism, leading to the stabilization of HIF1-α. This, in turn, triggers the transcriptional activation of WNT3, ultimately promoting YAP1-dependent hepatocyte proliferation and liver regeneration 138,139. GS converts glutamate and ammonia into glutamine 139. Notably, genetic deficiencies in either GS or the chaperone URI1 elevate circulating glutamate levels and consequently accelerate liver regeneration following partial hepatectomy 140. Glutamine is transported by the SLC1A5 transporter into liver cells, upregulates the expression of cell cycle genes, and promotes hepatocyte proliferation and liver regeneration 141. As a primary energy source for rapidly proliferating cells, glutamine is catabolized by glutaminase (GLS1/GLS2) to glutamate, which is further processed to generate α-KG. This metabolite replenishes the TCA cycle, furnishing both energy and biosynthetic precursors essential for liver regeneration 142. Following partial hepatectomy, glutamine contributes to ECM remodeling and exhibits spatially directed circulation within the liver parenchyma, mechanisms that collectively support the regenerative process 143. In addition, the glutamine metabolic enzyme GLS1 plays an important role in liver progenitor cell-mediated regeneration 25.

Alanine is indispensable for maintaining energy homeostasis during liver regeneration. As the primary substrate for hepatic gluconeogenesis, alanine helps to balance the energetic demands of glycogenolysis and regeneration, thereby maintaining systemic glucose homeostasis 144. Furthermore, alanine and other amino acids regulate insulin secretion and β-cell function via specific transporters, thereby indirectly supporting the systemic metabolic adaptations required for successful regeneration 145. Although significant progress has been made, a comprehensive understanding of how the amino acid metabolic network precisely coordinates the proliferation and differentiation of various hepatic cell populations during regeneration remains elusive. Consequently, therapeutic strategies aimed at modulating amino acid metabolism to enhance liver regeneration hold considerable promise for future clinical translation 143.

## 4. Application of amino acids in chronic liver disease

### 4.1 Diagnostic and prognostic biomarkers

Enzymes central to amino acid metabolism, notably aspartate aminotransferase (AST) and alanine aminotransferase (ALT), are well-established clinical biomarkers for liver injury. Beyond these enzymes, circulating amino acids themselves have emerged as promising biomarkers for the diagnosis, risk stratification, and prognosis assessment of a spectrum of liver diseases.

In MASLD, the serum amino acid profile exhibits characteristic dysregulation, with distinct alterations in specific amino acids carrying different pathological significance. Studies have shown that the levels of BCAAs in serum of patients with MASLD are significantly increased, and the changes in their concentration are associated with the improvement of liver fat content 146. Conversely, AAAs such as phenylalanine and tyrosine show positive associations with elevated fatty liver index (FLI) and framingham steatosis index (FSI), suggesting a potential contributory role in MASLD pathogenesis 147. In contrast, glycine, serine, threonine, and citrulline appear to exert protective effects, as their reduced circulating levels are associated with an increased risk of developing MASLD 148. The association of disordered amino acid metabolism with hepatic steatosis in cross-species animal models including geese, ducks, and rats further supports its role in human fatty liver 149,150,151. Metabolomic profiling has revealed that amino acids hold significant potential as diagnostic and prognostic markers for HCC. Their distinct alteration patterns offer a novel perspective for precise HCC identification and patient stratification. For example, the combination of arginine and histidine was identified as a highly effective diagnostic panel for HCC, achieving an area under the curve (AUC) of 0.998, with 96.7% sensitivity and 100% specificity 152. However, further validation in cohorts including patients with benign liver diseases is necessary to confirm its specificity in a clinically relevant differential diagnosis setting. A characteristic pattern of decreased serum levels of leucine, threonine, tryptophan, valine, and taurine, coupled with elevated phenylalanine, distinguishes HCC patients from healthy individuals 153. This specific amino acid signature can further differentiate HCC from patients with chronic hepatitis B (CHB). The results of transcriptome analysis show that HCC samples can be divided into different molecular subtypes based on the expression of amino acid metabolism-related genes AAMRGs, and these subtypes are significantly related to the prognosis of patients 154,155,156. In addition, serine and glycine metabolism are closely related to the tumor immune microenvironment (TIME) and prognosis of HCC, which can be used as potential prognostic markers 157. The serum amino acid profile undergoes progressive and characteristic changes during the transition from liver fibrosis to cirrhosis and ultimately to liver failure. Among these, the imbalance between BCAAs and tyrosine is particularly prominent and holds significant prognostic value. Studies have shown that the serum levels of branched-chain amino acids are significantly decreased and the levels of tyrosine are increased in patients with liver fibrosis and cirrhosis 13. Consequently, the resulting decline in the BCAA/tyrosine ratio serves as an independent predictor for the development of complications and poor overall prognosis in cirrhotic patients 158. This amino acid imbalance pattern is also prevalent in patients with liver failure, and the ratio is proposed as a potential biomarker for the prognosis of liver cirrhosis 13,131,132. The alterations in amino acid and related metabolite profiles across the spectrum of liver disease, are comprehensively summarized in Table 1.

Despite this promise, reliance on any single amino acid biomarker is insufficient for precise disease staging. In clinical practice, it is recommended to combine serum biomarkers with imaging techniques to improve diagnostic accuracy 159,160. In conclusion, while amino acid markers show considerable promise for risk stratification and prognostic assessment across various liver diseases, they are not yet sufficient as stand-alone tools for precise staging. A combination of serum markers and imaging techniques is still needed to improve diagnostic accuracy. In addition, certain drugs, such as metformin, may interfere with the predictive value of amino acid markers 161.

### 4.2 Therapeutic targets

Targeting amino acid metabolism provides a new strategy for the treatment of liver diseases. The intervention methods cover various aspects such as small molecule drugs, dietary supplements, and lifestyle modifications, and they act on the core mechanisms at different disease stages.

Therapeutic interventions that target critical nodes of amino acid metabolism can effectively reshape hepatic metabolic homeostasis. Glutamine serves as a major anaplerotic substrate for the hepatic TCA cycle, and its dysregulated metabolism is a key contributor to hepatic steatosis 48. For instance, preclinical studies indicate that in MASLD, dysregulation of GLS2 expression depletes TCA cycle intermediates, thereby redirecting metabolic flux toward gluconeogenesis and de novo lipogenesis 162. In animal models, the compound 3-thia fatty acid remodels the crosstalk between amino acid metabolism and the TCA cycle by inducing hepatic fatty acid oxidation and mitochondrial biogenesis 163. Similarly, berberine ameliorates dysregulated fatty acid and amino acid metabolic networks in both the heart and liver by modulating glycine, serine, and threonine metabolism 164. Sitagliptide combined with high-intensity interval training can synergistically improve the key pathways such as amino acid metabolism, bile acid metabolism and TCA cycle in diabetic mice 165. Dietary intervention is dependent on stages and background conditions, and it requires precise application. Increasing protein intake during weight loss reduces liver fat, but Western high-protein dietary patterns may exacerbate metabolic disorders, suggesting a bidirectional effect 166,167. The effects of BCAA vary depending on the stage of the disease. In early MASLD, elevated circulating BCAAs activate mTORC1 signaling, which in turn promotes de novo lipogenesis by upregulating sterol regulatory element-binding protein 1 (SREBP-1) 51. In contrast, clinical studies have shown that during the cirrhotic stage, BCAA supplementation can improve malnutrition and sarcopenia. Moreover, by enhancing BCAA catabolism and reducing the brain influx of neurotoxic aromatic amino acids, it helps restore the depressed Fischer's ratio, thereby offering benefit for preventing and treating hepatic encephalopathy 168,169.

Targeting amino acid metabolism has emerged as a pivotal direction in HCC therapy, primarily through mechanisms that directly inhibit tumor cell proliferation and remodel the immunosuppressive tumor microenvironment. Preclinical studies demonstrate that the GLS1 inhibitor CB-839 deprives HCC cells of glutamine, suppressing their proliferation by reducing the production of α-KG and the reducing equivalent NADPH 170. Research using HCC cell lines and animal models has found that methionine restriction or inhibition of MAT2A with FIDAS-5 induces cellular senescence in HCC and that combining these pro-senescence therapies with senolytic agents, such as GSK3 inhibitors that clear senescent cells, synergistically enhances antitumor efficacy 95,171. Mechanistic studies reveal that HCC cells disrupt the uptake of lysine by T cells, leading to impaired T cell immunity and thus escape immune surveillance 106. HCC cells upregulate SLC3A2 to enhance lysine uptake, leading to lysine depletion in the TME and impaired T cell cytotoxicity (defective granzyme B synthesis). Lysine supplementation restores T cell function and enhances the sensitivity of HCC to tyrosine kinase inhibitors and immune checkpoint blockade 172.

In the clinical management of chronic liver diseases, particularly cirrhosis and its complications, supplementation of specific amino acids has demonstrated significant therapeutic value. Their mechanisms of action encompass nutritional support, metabolic reprogramming, and direct anti-fibrotic or hepatoprotective effects. Clinical trials and management guidelines support the use of BCAA to improve malnutrition and sarcopenia in patients with liver cirrhosis 173. The decreased ratio of serum BCAAs to aromatic amino acids (Fischer's ratio) is associated with liver cirrhosis and HE, and BCAAs supplementation may help to improve this ratio 174. Experimental evidence from animal models of fibrosis suggests that L-serine plays an important role in anti-fibrosis therapy by regulating amino acid metabolism and reducing collagen deposition 127. Integrated multi-omics analyses in rodent models have revealed a protective role for taurine supplementation against liver fibrosis 175. In summary, targeting amino acid metabolism holds significant therapeutic potential for fatty liver, HCC, liver fibrosis, and complications of chronic liver disease. The underlying mechanisms are multifaceted, encompassing metabolic reprogramming, nutritional support, direct anti-fibrotic actions, detoxification, and immunomodulation 176,177. The active investigation of amino acid metabolism as a therapeutic target is evidenced by a growing number of clinical trials (summarized in Table 2), particularly focusing on pathways such as ammonia scavenging and de novo lipogenesis inhibition for advanced liver disease.

## 5. Discussion and future perspectives

This review synthesizes evidence underscoring a paradigm shift in our understanding of amino acids, recasting them from passive nutrients to active governors of the hepatic microenvironment. Their metabolism is intricately woven into the physiology of every hepatic cell type and is fundamentally reprogrammed across the spectrum of liver pathologies. From this synthesis, several overarching themes and critical future directions come to the fore.

### 5.1 The context-dependent duality of amino acid function

A central theme elucidated in this review is the profound context-dependency of amino acid actions. Their roles are not monolithic but exhibit remarkable metabolic plasticity, often demonstrating dualistic, and sometimes opposing, effects depending on the disease stage and cellular milieu.

Paradigm of Bidirectional Signaling: A prime illustration of this principle is the role of BCAAs. In early MASLD, elevated circulating BCAAs act as pathogenic drivers by activating mTORC1 signaling, thereby promoting hepatic de novo lipogenesis. Conversely, in advanced cirrhosis, BCAA supplementation serves a protective function, improving nutritional status and ameliorating hepatic encephalopathy. This duality extends to methionine, which can fuel tumor growth via MAT2A-mediated epigenetic reprogramming in HCC, yet also potentiate CD8⁺ T cell-mediated tumor killing by modulating the NR1I2/PCSK9 axis. Such profound complexity compels a departure from simplistic categorizations and demands a nuanced appreciation of amino acid flux and compartmentalization within the specific metabolic landscape of a given disease stage.

### 5.2 The networked view of amino acid metabolism: insights from multiplexed analyses

The amino acid metabolism disorder in liver diseases is not isolated but rather a complex and interrelated network. Changes in a single amino acid can affect the entire network through metabolic pathways, such as transamination and one-carbon metabolism. For instance, an increase in BCAAs and a decrease in glycine often occur simultaneously. Multimodal studies can reveal how they jointly drive hepatic steatosis and oxidative stress through the mTORC1 signaling pathway and glutathione synthesis pathway. Tumor cells support the TCA cycle and nucleotide synthesis through the “glutamine-serine” salvage reaction. Integrating multimodal data such as metabolomics, transcriptomics, and proteomics is key to analyzing this network.

The gut microbiome constitutes a critical, non-host component of the systemic amino acid metabolic network. For instance, integrative metabolomics and metagenomics have revealed that gut bacteria (e.g., Prevotella copri) can synthesize BCAAs, thereby directly influencing host circulating levels and insulin sensitivity. Conversely, microbial catabolism of dietary tryptophan not only competes with the host for this essential amino acid but also generates a spectrum of bioactive metabolites (e.g., indole derivatives, kynurenine). These metabolites can enter the portal circulation and signal to the liver through receptors like the aryl hydrocarbon receptor (AhR) or modulate enzyme activities, thereby reprogramming hepatic metabolic and inflammatory pathways. Therefore, a comprehensive understanding of the amino acid network in liver disease is incomplete without considering the gut microbiome as an integral metabolic entity.

### 5.3 Immunomodulatory dimensions of amino acid metabolism

The liver, functioning as a paramount immunometabolic organ, brings into sharp focus the critical crosstalk between amino acid availability and immune responses. It is now evident that amino acids serve as crucial immunometabolic checkpoints, actively shaping the tumor microenvironment and broader inflammatory landscape.

Amino Acids as Gatekeepers of Anti-Tumor Immunity: In HCC, tumoral overexpression of IDO1/TDO2 depletes tryptophan, generating kynurenine metabolites that activate the AhR pathway. This activation leads to suppression of CD8⁺ T cells and impairs the maturation of tertiary lymphoid structures. Similarly, tumor cell upregulation of the lysine transporter SLC3A2 creates a lysine-depleted microenvironment, crippling T cell function and cytotoxicity. These findings unequivocally position the control of specific amino acid availability as a fundamental mechanism of immune evasion. However, this immunometabolic interplay is complex. While L-methionine can potentiate T cell activity, global methionine restriction may indiscriminately compromise both tumor and immune cells. Therefore, the next generation of therapeutic strategies must achieve exquisite cellular selectivity. The goal is to precisely starve cancer cells of specific amino acids while preserving, or even augmenting, the metabolic fitness of immune effector cells—for instance, by targeting transporters or enzymes that are uniquely upregulated in malignant cells.

### 5.4 Technological frontiers for deeper mechanistic insight

A comprehensive understanding of the hepatic amino acid network demands the application of cutting-edge technologies that provide unprecedented resolution and dynamic data.

From Bulk Analysis to Spatial Resolution: While plasma amino acid profiles offer valuable biomarkers, they mask the significant heterogeneity among different hepatic cell types (e.g., periportal vs. pericentral hepatocytes, activated HSCs, various immune cell subsets). The integration of single-cell multi-omics (transcriptomics, metabolomics) with spatial transcriptomics and metabolomics will allow for the mapping of the “amino acid landscape” within the tissue architecture, revealing intricate cell-cell metabolic crosstalk and competition. This approach will unveil the intricate metabolic crosstalk, competition, and resource partitioning between neighboring cells.

Dynamic Flux Analysis and Causal Validation: Employing stable isotope tracing (e.g., with ¹³C-glutamine) in vivo or in ex vivo models enables the quantitative assessment of dynamic amino acid flux under pathological conditions, moving beyond static snapshots to understanding metabolic rewiring. Furthermore, leveraging CRISPR-Cas9 gene editingin advanced models like patient-derived organoids can establish causal relationships between specific amino acid metabolic genes and disease phenotypes. These technologies are pivotal for translating correlative observations into causative mechanistic insights.

### 5.5 Therapeutic Potential and the Path to Precision Medicine

Targeting amino acid metabolism holds significant therapeutic promise, but its translation requires sophisticated, combinatorial, and personalized approaches.

Rational Combination Therapies: Given the inherent redundancy and adaptability of metabolic networks, monotherapeutic targeting of a single amino acid pathway often invites compensatory resistance. The path forward lies in the rational design of intelligent combination strategies: (1) Vertical or Horizontal Pathway Combinations: Simultaneously targeting multiple nodes within a single pathway or parallel pathways (e.g., combining a GLS inhibitor with an xCT blocker to disrupt both energy metabolism and redox balance). (2) Metabolic-Immunologic Combinations: Pairing metabolic interventions with immunotherapy (e.g., combining IDO1 inhibition with immune checkpoint blockade to rescue exhausted T cells). (3) Targeting Adaptive Responses: Preemptively inhibiting compensatory survival pathways activated by amino acid deprivation (e.g., co-targeting autophagy or the ATF4-integrated stress response).

Precision Nutrition and Biomarker-Driven Stratification: Dietary interventions, including specific amino acid restriction or supplementation, represent a compelling and relatively low-risk adjunctive strategy. However, their effects are profoundly context-dependent and often bidirectional, as starkly illustrated by the stage-specific impact of BCAAs. Consequently, success hinges on biomarker-guided precision nutrition. The identification and validation of robust predictive biomarkers—such as tumoral ASS1 expression for stratifying patients for arginine depletion therapies, or specific serum amino acid ratios (e.g., BCAA/Tyr)—will be paramount. This will enable the tailoring of interventions to an individual's unique metabolic portrait and disease stage, decisively moving the field beyond a one-size-fits-all approach. Finally, personalizing amino acid-targeted interventions will necessitate a holistic view that incorporates the individual's gut microbiome composition, as it fundamentally influences host amino acid flux and the response to dietary or pharmacological modulations.

Furthermore, it is crucial to take into account the chronobiological factors. Currently, amino acids are used as biomarkers based on the “static” concentration measured from a single blood sample. However, amino acid metabolism is strongly regulated by circadian rhythms, dietary cycles, and sleep/wake cycles. Ignoring chronobiological factors may lead to an increase in the variability of biomarker measurements, thereby affecting the accuracy of diagnosis and prognosis. For instance, the amino acid profiles in the morning and evening may be significantly different. Understanding the diurnal rhythmic fluctuations in amino acid metabolism not only helps to optimize the sampling and interpretation of diagnostic biomarkers but may even pave the way for chronotherapy, which involves administering amino acid-targeted drugs at the most effective time of the day to maximize efficacy and minimize toxicity.

In conclusion, the study of amino acid metabolism has unveiled a sophisticated regulatory stratum that is fundamental to both hepatic homeostasis and pathogenesis. Amino acid functions are defined by three cardinal principles: profound context-dependent duality, deep integration with immune responses, and intricate spatial compartmentalization within the hepatic lobule. Future progress will be propelled by leveraging advanced technological frontiers to deconvolute this complexity at unprecedented resolution. By embracing, rather than simplifying, this intricate reality, we will undoubtedly unlock novel diagnostic modalities and pioneer a new class of precision therapeutic strategies that exploit the unique metabolic vulnerabilities of liver diseases. This journey from perceiving amino acids as simple nutrients to harnessing them as precise therapeutic targets epitomizes the transformative potential of metabolic medicine in modern hepatology.

## Figures and Tables

**Table 1:** Amino acids and related metabolites as diagnostic and prognostic biomarkers in MASLD, MASH, and HCC

Category	Disease Context	Change / Role	Related Metabolites/Pathways	Significance as Biomarker	Ref
BCAAs	MASLD/MASH	↑ early stage; ↓ with progression to cirrhosis	Branched-chain keto acids (BCKAs)	Correlates with insulin resistance and hepatic steatosis. Declining levels may indicate worsening liver function and progression.	77, 89,90
	HCC	Shifts associated with altered metabolic demands	mTORC1 signaling	May reflect reprogrammed energy metabolism supporting tumor growth.	136,137
AAAs	MASLD/MASH	↑ in plasma, further increasing with disease progression	Homocysteine	Indicates impaired hepatic clearance and is a marker of advancing liver injury and fibrosis.	77, 107
	HCC	Often elevated; linked to tumor metabolism and microenvironment	-	Sustained high levels may be associated with poor prognosis and tumor progression.	
Glutamine/Glutamate	MASLD/MASH	↑plasma Glutamate/Glutamine ratio	α-Ketoglutarate (αKG), Ammonia	Indicates mitochondrial dysfunction, oxidative stress, and HSC activation	79, 85, 86, 87
Glycine/Serine/Threonine	HCC	High consumption by tumor cells	TCA cycle anaplerosis	Supports rapid proliferation; tumor biomass and energy production.	
	MASLD/MASH	↓ in plasma	Glutathione, SAMe, One-carbon metabolism	Low levels impair antioxidant defense (reduced GSH) and contribute to lipotoxicity and oxidative stress.	73, 97, 98
	HCC	Demand increased for nucleotide synthesis and methylation	Purines, dTMP, SAMe	Supports uncontrolled cell proliferation and epigenetic alterations in tumors.	
Sulfur-Amino Acids	MASLD/MASH	Dysregulated cycle; ↑ Homocysteine	SAMe, SAH, Glutathione	Hyperhomocysteinemia promotes oxidative stress, inflammation, fibrosis, and impaired VLDL secretion.	14, 107, 108
	HCC	Altered methionine metabolism	SAMe, Polyamines	Methionine restriction can suppress tumor growth	105, 144
Tryptophan	MASLD/MASH	Levels influenced by gut microbiota	Indolepropionic acid (IPA), Kynurenine	Microbiota-derived IPA strengthens gut barrier; shift to kynurenine pathway may promote immune tolerance.	125,128,129
	HCC	Altered metabolism in tumor microenvironment	Kynurenine	Increased kynurenine can suppress anti-tumor immunity, facilitating immune evasion.	

**Table 2:** Clinical trials of drugs targeting amino acid metabolism

Drug Name	Target Amino Acid	Development Phase	Outcomes	Clinical Trial ID
Arginine hydrochloride	Arginine	PHASE3	Treatment of Advanced Hepatocellular Carcinoma	NCT03950518
S-adenosyl-L-methionine (SAMe)	Methionine	PHASE3	Treatment of Nonalcoholic Fatty Liver Disease	NCT01754714
Branch Chain Amino Acid	Branched-chain Amino Acids (Leucine, Isoleucine, Valine)	PHASE2	Supplementation for Hepatocellular Carcinoma	NCT03908255
Arginine hydrochloride	Arginine	PHASE3	Treatment of Intermediate-stage Hepatocellular Carcinoma	NCT03274427
DRP-104	Glutamine	PHASE1|PHASE2	Treatment of Advanced Stage Fibrolamellar Carcinoma	NCT06027086
Siliphos-Selenium-Methionine-Alpha Lipoic Acid	Methionine	PHASE4	Treatment of Fatty Liver and Non-alcoholic Steatohepatitis	NCT01650181
S-adenosylmethionine	Methionine	PHASE3	Therapy for Non-Alcoholic Steatohepatitis	NCT00108589

## Abbreviations

MASLD: metabolic dysfunction-associated steatotic liver disease
HCC: hepatocellular carcinoma
4-HPD: 4-hydroxyphenylpyruvate dioxygenase
AAAs: aromatic amino acids
BCAAs: branched-chain amino acids
EECs: enteric endocrine cells
NPCs: Non-parenchymal cells
HSCs: hepatic stellate cells
GLS1: glutaminase 1
HGF: hepatocyte growth factor
system xc-: cystine/glutamate antiporter
ATF4: Activating transcription factor 4
ISR: integrated stress response
ASS: argininosuccinate synthase
MASH: metabolic dysfunction-associated steatohepatitis
NASH: non-alcoholic steatohepatitis
FGF21: fibroblast growth factor 21
GSH: glutathione
GNMT: glycine N-methyltransferase
SAMe: S-adenosylmethionine
SREBP: sterol regulatory element-binding protein
SHMT2: serine hydroxymethyltransferase 2
HFD: high-fat diet
3-PG: 3-phosphoglycerate
TCA: tricarboxylic acid
GLS2: glutaminase 2
GS: glutamine synthetase
ACC: acetyl-CoA carboxylase
GLS: glutaminase
GLUD: glutamate dehydrogenase
GOT2: glutamate-oxaloacetate transaminase 2
CCL2: C-C motif chemokine ligand 2
ECM: extracellular matrix
EMT: epithelial-mesenchymal transition
MAT2A: methionine adenosyltransferase 2A
ASS1: Argininosuccinate synthase 1
PRMT1: protein arginine methyltransferase 1
IDO1: indoleamine 2,3-dioxygenase 1
TDO2: tryptophan 2,3-dioxygenase
Kyn: kynurenine
AhR: aryl hydrocarbon receptor
TLS: tertiary lymphoid structure
TME: tumor microenvironment
GPX4: glutathione peroxidase 4
ACLF: acute-on-chronic liver failure
GRβ: glucocorticoid receptor β
α-KG: α-ketoglutarate
HSCs: hepatic stellate cells
NMDA: N-methyl-D-aspartate
AST: aspartate aminotransferase
ALT: alanine aminotransferase
FLI: fatty liver index
AUC: area under the curve
CHB: chronic hepatitis B
TIME: tumor immune microenvironment
SREBP-1: sterol regulatory element-binding protein 1
